# Association between types of surgery and cancer-specific death in patients with early differentiated thyroid carcinoma: a real-world study

**DOI:** 10.3389/fonc.2025.1540705

**Published:** 2025-07-16

**Authors:** Jia-wei Yu, Rui Pang, Bo Liu, Liang Zhang, Ling-yu Kong

**Affiliations:** Department of Head and Neck Thyroid, Harbin Medical University Cancer Hospital, Harbin, Heilongjiang, China

**Keywords:** differentiated thyroid carcinoma, surgery, outcome, SEER, competitive risk model

## Abstract

**Objective:**

To explore the association between types of surgery and outcomes in patients with early differentiated thyroid carcinoma (DTC) by a real-world study.

**Methods:**

All the data were from Surveillance, Epidemiology, and End Results (SEER). Types of surgery included Surgery 1 (lobectomy, isthmectomy, or removal of less than a lobe), Surgery 2 (subtotal or near total thyroidectomy, or removal of a lobe and partial removal of the contralateral lobe), and Surgery 3 (total thyroidectomy). The association between types of surgery and DTC or other causes of death was explored by a competitive risk model and subgroup analysis. We also used the machine learning algorithm to evaluate the importance of types of surgery on long-term outcomes.

**Results:**

A total of 7,230 patients were enrolled, of whom 1,512, 249, and 5,469 patients received Surgery 1, Surgery 2, and Surgery 3, respectively. The long-term outcomes among the three groups differed significantly (P < 0.001). Competitive risk analysis showed that types of surgery were significantly associated with DTC-related death (P=0.005), other causes death (P<0.001) in the crude model, and three adjusted models further indicated their independent association (all adjusted P<0.05). Specifically, Surgery 2 was associated with the highest DTC-related death. However, when the death of other causes was refined, types of surgery were only related to DTC-related death (all adjusted P<0.05). The importance analysis suggested that the impact of surgical type on long-term outcomes may be underrecognized.

**Conclusions:**

The types of surgery were significantly related to the DTC-related death of patients, and it deserved attention. Additionally, Surgery 2 was associated with higher DTC-related death.

## 1 Introduction

Differentiated thyroid carcinoma (DTC), including papillary thyroid carcinoma (PTC) and follicular thyroid carcinoma (FTC), represents a common thyroid malignancy, accounting for 90% of thyroid cancers (1, 2). Over the past few decades, the incidence of DTC has increased globally (3). Among the annually diagnosed thyroid cancer (TC) patients, DTC comprises 95%, with PTC alone accounting for over 88% (4). Although DTC demonstrates relatively low malignant potential and mortality rates, its potential for recurrence and treatment-related complications necessitates standardized therapeutic management and lifelong surveillance to mitigate long-term health impacts and preserve quality of life (5).

Early-stage DTC patients generally present with benign thyroid nodules. Surgical intervention is considered when patients exhibit clinical symptoms of significant local compression related to the nodule, signs of malignant transformation, tumors located in the substernal or mediastinal regions, hyperthyroidism with high-functioning adenomas (TA), or toxic multinodular goiter (TMNG) (6).

The fundamental principle in the surgical treatment of benign thyroid nodules is to safely excise the target lesions while preserving as much normal thyroid tissue as possible, depending on the circumstances. For instance, if nodules are distributed bilaterally, making it challenging to retain substantial normal thyroid tissue, total or near-total thyroidectomy is typically chosen. In cases of TMNG, common procedures include bilateral lobectomy, unilateral lobectomy, isthmectomy, and subtotal lobectomy (7). Additionally, intraoperative precautions are crucial to protect the parathyroid glands, recurrent laryngeal nerve, and superior laryngeal nerve. In short, the choice of surgical method significantly impacts the patient’s prognosis and quality of life (8–10).

Despite the established surgical principles, the relationship between surgical techniques and the prognosis of DTC patients remains unclear. This study aims to explore this relationship using data from the SEER database. We employed competitive risk models to analyze the relationship between surgical methods and prognosis in early-stage DTC patients and investigated the association between several significant DTC-related deaths and other causes of death.

## 2 Method

### 2.1 Data source and study population

This study utilized SEERStat software (version 8.3.6) to retrieve medical records of 114,911 patients diagnosed with DTC from 2010 to 2019 within the SEER database. The ICD codes for DTC included 8050/3, 8260/3, 8330/3, 8331/3, 8332/3, 8335/2, 8339/3, 8340/2, 8340/3, 8341/2, 8341/3, 8342/3, 8343/2, 8343/3, and 8344/3. We next excluded patients who received radiotherapy (n=48,995); patients with unknown staging or stage 3 or 4 (n=34,748); patients with T0 and TX stages (n=599); patients who did not undergo surgical treatment (n=737); patients with surgery codes 13 (Local tumor destruction), 80 (Thyroidectomy, NOS), 90 (Surgery, NOS), and 99 (Unknown if surgery performed) (n=209); patients with unknown bone metastasis status (n=82); patient with brain metastasis (n=1); patient with unknown liver metastasis status (n=1); patients with unknown lung metastasis status (n=8); patients diagnosed by non-histological methods (only Positive histology included) (n=368); patients with unknown tumor size (n=336); patients with NX stage (n=109); patients who received chemotherapy (n=32); patients with low differentiation or unknown differentiation status (n=21,437); patients aged 90+(n=3); patients with unknown cause of death (n=12); patients with M1 stage (n=4). Ultimately, 7,230 patients were included in the study. All included patients were those who underwent surgical treatment alone, were classified as stage 1 or 2, and had no distant metastasis.

### 2.2 Study variables

We gathered demographic characteristics and clinical indicators for the DTC patients included in the study. These variables encompass a comprehensive range of patient information. Specifically, the variables include: age, gender, race, marital status, median household income, county type, grade, histologic, clinical stage, T stage, N stage, multifocality, neck dissection, number of lymph nodes removed, surgical treatment methods, treatment delay days, tumor size (cm), positive lymph node numbers (PLNNs), examined lymph node number (ELNN), and long-term outcomes (survival time, vital status, causes of death). Notably, the surgical treatment methods are classified into three types according to the study methodology of Cao et al. (11). Surgery 1: lobectomy, isthmectomy, or removal of less than a lobe. Surgery 2: subtotal or near total thyroidectomy, or removal of a lobe and partial removal of the contralateral lobe (subtotal thyroidectomy: affected thyroid gland lobe (including capsule) + thyroid isthmus + thyroid vertebral lobe + contralateral partial gland lobe; near total thyroidectomy: almost all thyroid tissues were removed, and about 1 gram of normal tissue was retained on each side to protect parathyroid gland and recurrent laryngeal nerve). Surgery 3: total thyroidectomy (complete removal of all thyroid tissue).

The causes of death were categorized into two main types: cancer-specific death and non-specific death. Within the non-specific causes, we focused on several significant categories: cardiovascular diseases (as per the SEER database), infectious diseases, respiratory diseases, and gastrointestinal diseases. Following the methodology outlined in the study by Wang et al. (12), the causes of death recorded in the SEER database were classified as follows: Cardiovascular diseases: causes labeled as aortic aneurysm and dissection, cerebrovascular diseases, diseases of the heart, hypertension without heart disease, and other diseases of arteries or arterioles capillaries. Infectious diseases: causes labeled as pneumonia and influenza, septicemia, and other infectious and parasitic diseases including human immunodeficiency virus (HIV). Respiratory diseases: causes labeled as lung and bronchus, chronic obstructive pulmonary disease, and Allied Cond. Gastrointestinal diseases: causes labeled as colon excluding rectum, stomach, and chronic liver disease and cirrhosis.

### 2.3 Statistical analysis

Data processing and analysis were performed using SPSS statistical analysis software. Categorical data were expressed as numbers and percentages (%), and comparisons between different surgical groups were conducted using the Chi-square test. Continuous variables were analyzed by one-way analysis of variance (ANOVA) and were expressed as median [25%, 75%]. In the competing risks model, DTC-related death was considered the event of interest, while death from other causes was treated as competing events. Survival was treated as a censored event. The Fine-Gray method was used for statistical testing in the competing risks model. This analysis was implemented using the “cmprsk” package in R software. Different variables were adjusted in the various risk models (specific model adjustments can be copied from the original text). Three methods including Logistic, random forest, and XGBoost were then used to rank the importance of variables to explore the contribution of surgical approaches to long-term survival in patients. P < 0.05 indicated that the difference was statistically significant.

## 3 Results

### 3.1 The baseline data of patients with DTC

In this study, a total of 7,230 patients were enrolled. Those patients only received the surgical treatment (without radiotherapy or chemotherapy), were in the early stage of DTC, and had no distant metastasis. Among them, 1,512 patients received Surgery 1 (lobectomy, isthmectomy, or removal of less than a lobe); 249 patients received Surgery 2 (subtotal or near total thyroidectomy, or removal of a lobe and partial removal of the contralateral lobe); and 5,469 patients received Surgery 3 (total thyroidectomy). Except for the race, marital status, clinical stage, and multifocality, the remaining variables all showed significant differences among three surgery subgroups (**Table 1**, all P<0.05). The long-term outcomes among three groups were different (P<0.001).

**Table 1:** The baseline data of patients with early DTC grouped by types of surgery.

Variables	Subgroups	Surgery 1 (n=1512)	Surgery 2 (n=249)	Surgery 3 (n=5469)	P
Age (years)		52 [40, 63]	51 [38, 60]	48 [37, 59]	<0.001
Gender	male	390 (25.794)	51 (20.482)	1010 (18.468)	<0.001
	female	1122 (74.206)	198 (79.518)	4459 (81.532)	
Race	White	1239 (81.944)	193 (77.510)	4398 (80.417)	0.184
	Non-White	273 (18.056)	56 (22.490)	1071 (19.583)	
Marital status	M1	290 (20.308)	58 (24.473)	1224 (23.529)	0.070
	M2	962 (67.367)	149 (62.869)	3411 (65.571)	
	M3	176 (12.325)	30 (12.658)	567 (10.900)	
Median household income	<$80,000	904 (59.788)	177 (71.084)	3392 (62.022)	0.003
	≥$80,000	608 (40.212)	72 (28.916)	2077 (37.978)	
County type	nonmetropolitan	189 (12.508)	53 (21.285)	535 (9.782)	<0.001
	metropolitan	1322 (87.492)	196 (78.715)	4934 (90.218)	
Grade	grade I	1386 (91.667)	220 (88.353)	4834 (88.389)	0.001
	grade II	126 (8.333)	29 (11.647)	635 (11.611)	
Histologic	PTC	1422 (94.048)	240 (96.386)	5328 (97.422)	<0.001
	FTC	90 (5.952)	9 (3.614)	141 (2.578)	
Clinical stage	I	1425 (94.246)	231 (92.771)	5171 (94.551)	0.460
	II	87 (5.754)	18 (7.229)	298 (5.449)	
T stage	T1	1303 (86.177)	203 (81.526)	4523 (82.703)	<0.001
	T2	170 (11.243)	36 (14.458)	651 (11.903)	
	T3+T4	39 (2.579)	10 (4.016)	295 (5.394)	
N stage	N0	1495 (98.876)	241 (96.787)	5034 (92.046)	<0.001
	N1	17 (1.124)	8 (3.213)	435 (7.954)	
Multifocality	solitary	193 (12.765)	25 (10.040)	600 (10.971)	0.122
	multifocal	1319 (87.235)	224 (89.960)	4869 (89.029)	
Neck dissection	no	1085 (72.189)	168 (68.016)	2781 (51.225)	<0.001
	yes	418 (27.811)	79 (31.984)	2648 (48.775)	
Lymph nodes removed	none	1085 (72.285)	168 (68.571)	2781 (51.740)	<0.001
	1-3	345 (22.985)	54 (22.041)	1567 (29.153)	
	≥4	71 (4.730)	23 (9.388)	1027 (19.107)	
Treatment delay days	≤30	1294 (86.497)	213 (87.295)	3873 (73.394)	<0.001
	>30	202 (13.503)	31 (12.705)	1404 (26.606)	
Tumor size (cm)	<1	1022 (67.593)	137 (55.020)	2834 (51.819)	<0.001
	1-2	290 (19.180)	70 (28.112)	1800 (32.913)	
	>2	200 (13.228)	42 (16.867)	835 (15.268)	
ELNN	0	1075 (71.098)	169 (67.871)	2741 (50.119)	<0.001
	1-4	379 (25.066)	57 (22.892)	1807 (33.041)	
	>4	58 (3.836)	23 (9.237)	921 (16.840)	
PLNNs	no	420 (27.778)	73 (29.317)	2282 (41.726)	<0.001
	yes	1092 (72.222)	176 (70.683)	3187 (58.274)	
Long-term outcomes	censoring event	1402 (92.725)	224 (89.960)	5209 (95.246)	<0.001
	interested event	4 (0.265)	4 (1.606)	18 (0.329)	
	competitive event	106 (7.011)	21 (8.434)	242 (4.425)	

Types of surgery are divided into three categories: Surgery 1 (Lobectomy, Isthmectomy, or Removal of Less Than a Lobe); Surgery 2 (Subtotal or Near Total Thyroidectomy, or Removal of a Lobe and Partial Removal of the Contralateral Lobe); Surgery 3 (Total Thyroidectomy). Marital status: M1, Single (never married)+Separated+ Unmarried or Domestic Partner;M2, Married (including common law); M3, Divorced+Widowed. PLNNs, positive lymph node numbers; ELNN, examined lymph node number. T stage: The sample size of patients with T4 was small, therefore the T3 and T4 were combined. Outcomes: interested event (DTC related death); competitive event (other causes death); censoring event (patients who were lost before interested events and competition events occurred, and patients who had neither interested events nor competitive events after follow-up).

### 3.2 Association between types of surgery and long-term outcomes

We then explored the association between the types of surgery and long-term outcomes in patients with DTC by competitive risk model. The univariable analysis showed that types of surgery were significantly related to DTC-related death (stat=10.706, P=0.005). It was also associated with the other causes death of patients (stat=21.310, P<0.001). These results showed the importance of types of surgery on the long-term outcomes of patients with DTC.

Next, we performed the multivariable competitive risk model analysis to explore the independent association between the types of surgery and long-term outcomes (**Table 2**). The results indicated that Surgery 2 was related to a higher risk of DTC-related death in adjusted model 1 (HR=7.966, P=0.003), model 2 (HR=6.242, P=0.012), and model 3 (HR=6.198, P=0.010). However, Surgery 3 was related to a lower risk of other causes death in adjusted model 1 (HR=0.786, P=0.037), model 2 (HR=0.729, P=0.008), and model 3 (HR=0.721, P=0.006). In addition, age, gender, and N stage were the common independent factors of DTC and other causes death (all P<0.05, data not shown).

**Table 2:** Association analysis between types of surgery and long-term outcomes by competitive risk model.

Model	Surgery type	DTC related death		Other causes death	
		HR (95%CI)	P	HR (95%CI)	P
Model 1	Surgery 1	Reference		Reference	
	Surgery 2	7.966 [2.010, 31.564]	0.003	1.208 [0.769, 1.899]	0.410
	Surgery 3	1.807 [0.610, 5.351]	0.290	0.786 [0.626, 0.985]	0.037
Model 2	Surgery 1	Reference		Reference	
	Surgery 2	6.242 [1.491, 26.140]	0.012	1.183 [0.741, 1.889]	0.480
	Surgery 3	1.569 [0.500, 4.926]	0.440	0.729 [0.577, 0.919]	0.008
Model 3	Surgery 1	Reference		Reference	
	Surgery 2	6.198 [1.535, 25.021]	0.010	1.121 [0.697, 1.805]	0.640
	Surgery 3	1.407 [0.448, 4.419]	0.560	0.721 [0.571, 0.910]	0.006

HR, hazard ratio; CI, confidence intervals. Surgery 1 (Lobectomy, Isthmectomy, or Removal of Less Than a Lobe); Surgery 2 (Subtotal or Near Total Thyroidectomy, or Removal of a Lobe and Partial Removal of the Contralateral Lobe); Surgery 3 (Total Thyroidectomy).

Model 1: adjusted for the age, gender, median household income, and county type.

Model 2: adjusted for the grade, histologic, T stage, N stage, tumor size, positive lymph node numbers, and examined lymph node number.

Model 3: adjusted for the neck dissection, lymph nodes numbers removed, and treatment delay days.

**Figure 1:**
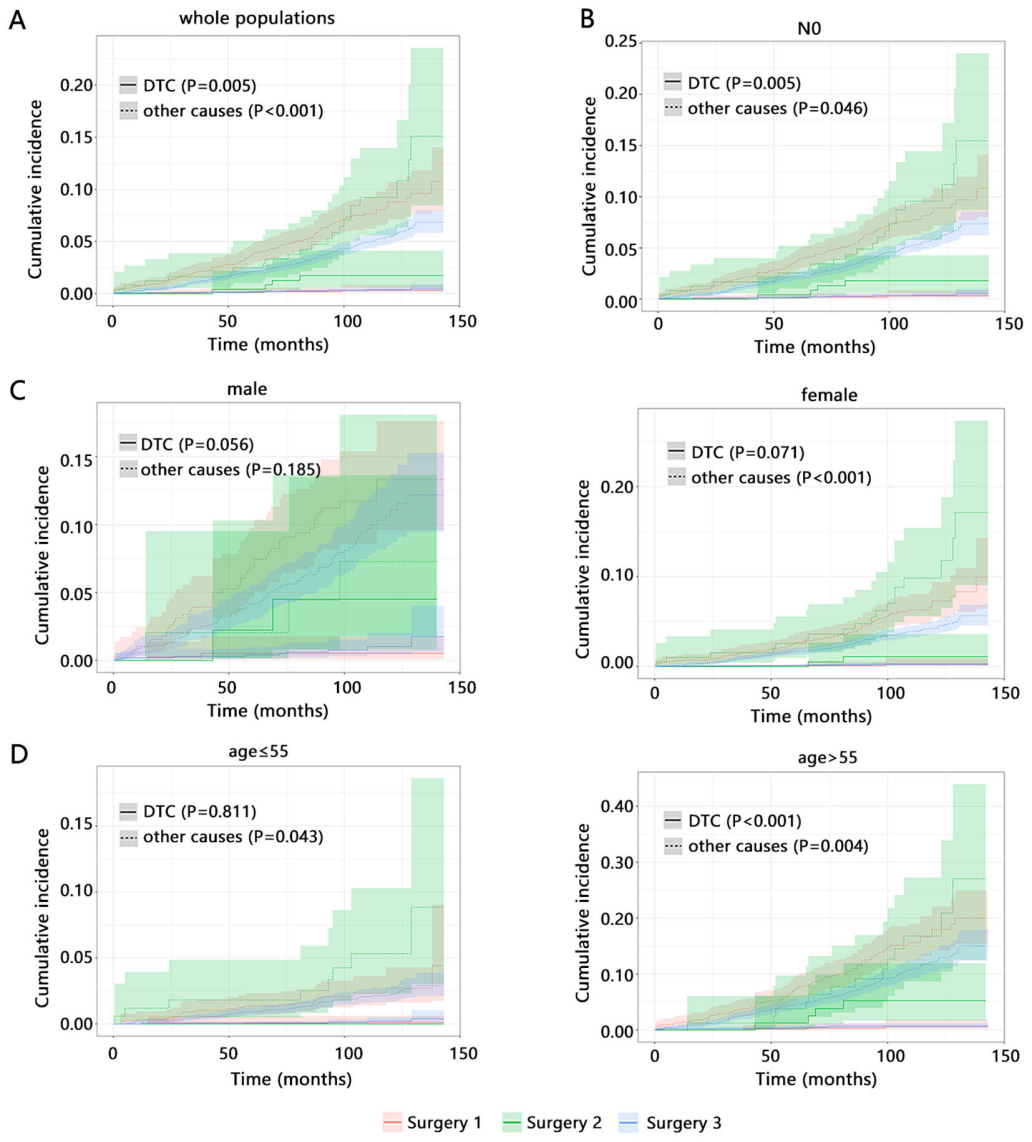
The influence of types of surgery on the cumulative incidence of long-term outcomes by competitive risk analysis. **(A)** whole populations. Subgroup populations with **(B)** N0 stage. The samples size of patients with N1 did not support the analysis. **(C)** different age. **(D)** different gender. Surgery 1 (Lobectomy, Isthmectomy, or Removal of Less Than a Lobe); Surgery 2 (Subtotal or Near Total Thyroidectomy, or Removal of a Lobe and Partial Removal of the Contralateral Lobe); Surgery 3 (Total Thyroidectomy).

We subsequently explored the influence of types of surgery on the cumulative incidence rate of DTC and other causes death. Among whole populations (**Figure 1A**), Surgery 2 caused the highest cumulative incidence rate of DTC-related death while Surgery 3 caused the lowest other causes death, which were consistent with the results obtained from the above multivariable competitive risk analysis. Age, gender, and N stage were the common independent factors of DTC and other causes death. Therefore, we also explored the influence of types of surgery on the long-term outcomes among these subgroups. Its influence in patients with N0 stage and these patients with age>55 years old (**Figures 1B, D**) was similar with that of whole populations. However, among female patients (**Figure 1C**, P<0.001) and those with age ≤ 55 years old (**Figure 1D**, P=0.043), types of surgery were only related to the other causes death and Surgery 2 caused the highest cumulative incidence rate of other causes death.

**Figure 2:**
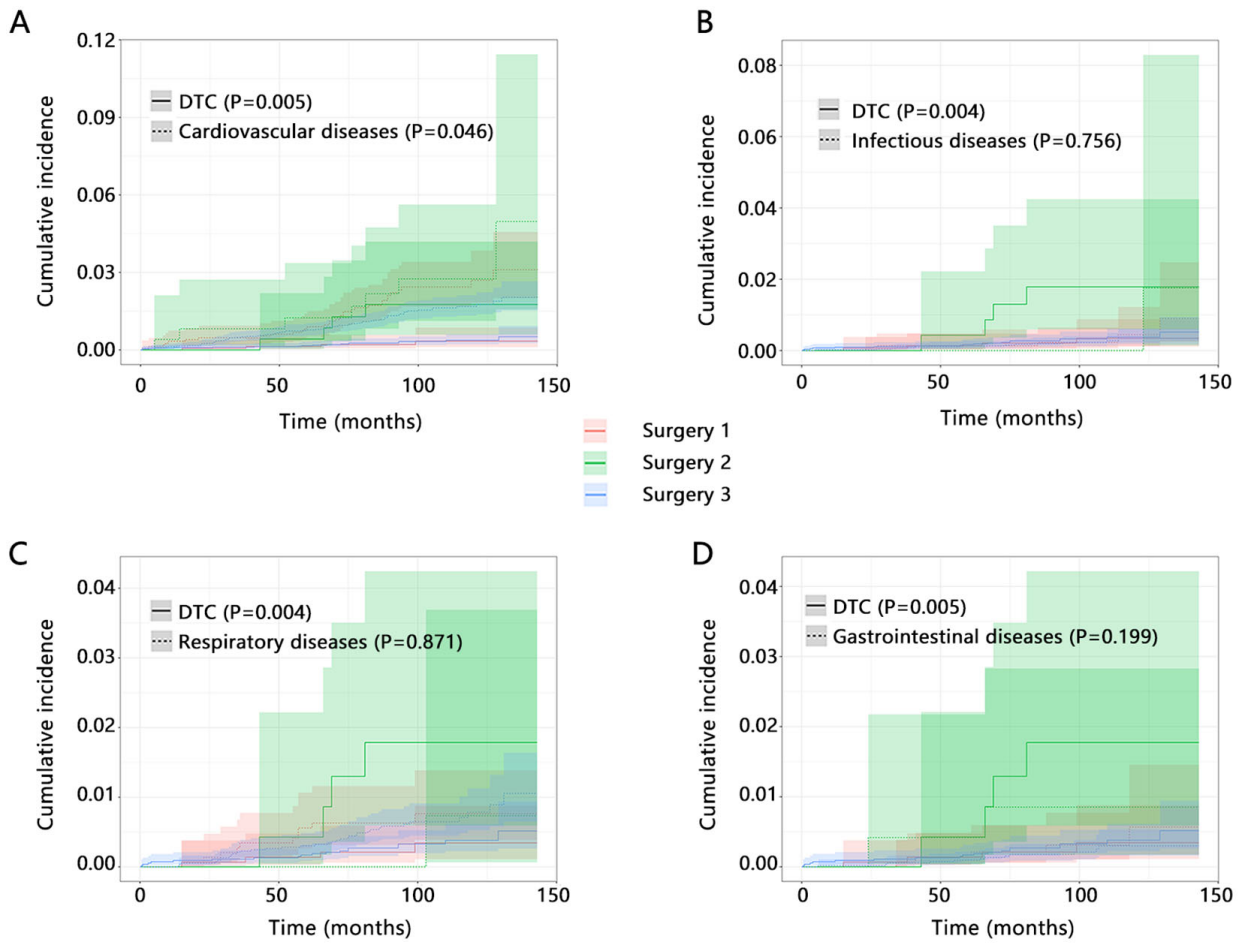
The influence of types of surgery on the cumulative incidence of DTC-related death and other causes death by competitive risk analysis. Interested event was DTC-related death, and competitive event was set as **(A)** Cardiovascular diseases related death; **(B)** Infectious diseases related death; **(C)** Respiratory diseases related death; and **(D)** Gastrointestinal diseases related death. Surgery 1 (Lobectomy, Isthmectomy, or Removal of Less Than a Lobe); Surgery 2 (Subtotal or Near Total Thyroidectomy, or Removal of a Lobe and Partial Removal of the Contralateral Lobe); Surgery 3 (Total Thyroidectomy).

Further, we refined the death of other causes to four categories, including cardiovascular diseases related death, infectious diseases related death, respiratory diseases related death, and gastrointestinal diseases related death. We explored the influence of types of surgery on the cumulative incidence of DTC-related death and four types of causes death by competitive risk analysis. The results showed that types of surgery can significantly influence the cumulative incidence of DTC-related death no matter what kind of disease was set as its competitive event (**Figures 2A-D**, all P<0.05). However, our result also showed potential influence of types of surgery on the cardiovascular diseases related death (P=0.046), which suggested the importance of types of surgery on the cardiovascular diseases related death.

We next performed a multivariable competitive risk model analysis to explore the independent association between the types of surgery and DTC or cardiovascular diseases related death (**Table 3**). When cardiovascular diseases related death was detailly set as the competitive event of DTC-related death, the types of surgery were still related to the DTC-related death both in model 1 (HR=7.765, P=0.004) and model 2 (HR=6.186, P=0.012). However, the independent association between types of surgery and cardiovascular diseases related death was not observed after adjusting for several variables (all P>0.05). These results further highlighted the importance of types of surgery on the DTC-related death and their stable association.

**Table 3:** Association between types of surgery and outcomes by multivariable competitive risk analysis.

Model	Surgery type	DTC related death		Cardiovascular diseases related death	
		HR (95%CI)	P	HR (95%CI)	P
Model 1	Surgery 1	Reference		Reference	
	Surgery 2	7.765 [1.965, 30.689]	0.004	1.417 [0.628, 3.195]	0.400
	Surgery 3	1.787 [0.604, 5.285]	0.290	0.883 [0.596, 1.310]	0.540
Model 2	Surgery 1	Reference		Reference	
	Surgery 2	6.186 [1.481, 25.845]	0.012	1.309 [0.575, 2.979]	0.520
	Surgery 3	1.550 [0.494, 4.862]	0.450	0.786 [0.515, 1.201]	0.270

HR, hazard ratio; CI, confidence intervals. Surgery 1 (Lobectomy, Isthmectomy, or Removal of Less Than a Lobe); Surgery 2 (Subtotal or Near Total Thyroidectomy, or Removal of a Lobe and Partial Removal of the Contralateral Lobe); Surgery 3 (Total Thyroidectomy).

Model 1: adjusted for the age, gender, median household income, and county type.

Model 2: adjusted for the grade, histologic, T stage, N stage, tumor size, positive lymph node numbers, and examined lymph node number.

The sample size in Model 3 did not support the statistical analysis.

### 3.3 Influence of types of surgery on the long-term outcomes of patients may be easily overlooked

Above results have demonstrated significant association between types of surgery and long-term outcomes of patients with DTC. We finally evaluated the importance degree of types of surgery among all variables by 3 machine learning algorithms based on Logistic, Random forest, and XGBoost methods (**Table 4**). Among top 5 variables from 3 algorithms, gender was the common factor, implying the importance of gender on the long-term outcomes of patients. However, the ranking of the types of surgery was not observed among the top 10 variables. The results highlight the potential for surgical modality to be neglected in longitudinal assessments of patient prognosis.

**Table 4:** The importance ranking of variables on the long-term outcomes by machine learning algorithms.

Logistic	Random forest	XGBoost
Gender (0.57)	Age (0.67)	Age (2961)
Histologic (0.184)	Tumor size (0.062)	Tumor size (633)
ELNN (0.174)	Treatment delay days (0.04)	Gender (462)
PLNN (0.151)	Gender (0.039)	Median household income (389)
T stage (0.148)	Median household income (0.037)	Treatment delay days (374)
County type (0.095)	Grade (0.033)	T stage (327)
Age (0.081)	County type (0.032)	County type (300)
Median household income (0.038)	T stage (0.023)	Grade (289)
Tumor size (0.008)	Histologic (0.020)	ELNNs (288)
Grade (0.001)	Lymph nodes removed (0.016)	Neck dissection (232)

ELNN, examined lymph node number; PLNN, positive lymph node numbers.

## 4 Discussion

This study explored the association between types of surgery and long-term outcomes of patients with DTC. We found that patients with Surgery 2 had the highest risk of DTC-related death, while patients with Surgery 3 had the lowest risk of other causes of death. However, after refining the death of other causes, types of surgery were only related to the DTC-related death.

Compared with Surgery 1 and Surgery 3, Surgery 2 was associated with a higher risk of DTC-related death in this study, which may be explained through multi-tiered pathophysiological mechanisms. First, regarding postoperative thyroid remnant tissue, in Surgery 1, when lesions are confined to a single lobe and the isthmus, complete removal of the affected lobe or isthmus results in an extremely low probability of residual lesions. In the absence of lymphatic invasion, there is effectively no residual tissue, rendering the recurrence probability virtually zero. In Surgery 3, all lesion foci are removed, eliminating the source of potential recurrence. However, compared to Surgery 1 and Surgery 3, Surgery 2 carries the highest risk of residual tumor tissue within the remaining thyroid tissue. Furthermore, existing research demonstrates that the mass of the thyroid remnant in Surgery 2 is positively correlated with the recurrence rate (13, 14). Residual thyroid tissue may harbor occult microcarcinomas, particularly in multifocal disease. Surgical trauma-induced inflammatory microenvironments stimulate VEGF-mediated angiogenesis, enhancing proliferation and metastatic potential of residual cancer cells while selecting for aggressive clones that directly promote disease progression (15). Second, remnant tissue compromises postoperative surveillance: persistently secreted thyroglobulin (Tg) obscures serum Tg levels as a tumor burden biomarker, reducing sensitivity even during stimulated Tg testing. Concurrently, competitive radioiodine uptake by residual tissue causes false-negative whole-body scans, collectively delaying recurrence diagnosis (16). Third, in patients requiring adjuvant radioiodine therapy (RAI), remnants both dilute radiation doses to malignant foci and suppress TSH via negative feedback, downregulating sodium-iodide symporter (NIS) expression and rendering RAI ineffective (17). Thyroid cancer surgery carries a risk of damaging the parathyroid glands and the recurrent laryngeal nerve (RLN). RLN injury can lead to recurrent aspiration pneumonia, potentially worsening the prognosis for patients with comorbidities. Compared to Surgery 1 and Surgery 3, recurrence from residual lesions in Surgery 2 often necessitates reoperation. During such secondary surgery, the risk of RLN injury and permanent hypoparathyroidism increases by 2–10 times compared to the initial surgery (18–21). This is primarily due to scar tissue left from the first surgery. Transient RLN palsy occurs in up to 15-23% of cases, while permanent injury occurs in 2.6-15.5% (22, 23). The fibrotic tissue causing anatomical distortion leads to a sharp increase in the risk of RLN injury and permanent hypoparathyroidism during reoperation. Overall, this triad of escalating morbidity, compromised treatment efficacy, and accelerated cancer advancement collectively elevates DTC-related death in patient with Surgery 2.

Our study also found that compared to lobectomy, isthmectomy, or less extensive thyroidectomy, total thyroidectomy is associated with the lowest risk of other causes death mortality. This may be due to the total thyroidectomy avoiding the complications associated with incomplete thyroidectomy, thus reducing other causes death mortality risk (24–26).

Through subgroup analysis, we discovered that in females or patients under 55 years old, Surgery 2 is associated with the highest risk of other causes death mortality. This may be because these groups typically have longer life expectancies, making them more susceptible to the long-term risks of such surgeries (recurrence, limited effectiveness of RAI therapy in high-risk patients, surgical complications, etc.) (26, 27). Additionally, females are more prone to endocrine disorders and osteoporosis post-surgery and treatment, which can affect overall health and other causes death mortality (28).

This study has several limitations. We excluded patients who underwent radiotherapy or chemotherapy, focusing solely on the correlation between surgical methods and patient survival among surgical patients. Therefore, it is unclear whether radiotherapy or chemotherapy affects this correlation. Additionally, to avoid confounding factors, we excluded patients with metastasis, even though metastasis significantly impacts prognosis. It remains uncertain whether metastasis and surgical methods collectively influence patient outcomes. Future studies should further investigate these issues. The SEER database does not include recurrence as a variable, which can affect clinical outcomes. Furthermore, we were unable to adjust for some potential confounders, such as patient comorbidities, the level of the hospital that affects the operation, because these data were not currently included in the SEER database.

## 5 Conclusions

Our study indicated the significant association between types of surgery and DTC-related death. Particularly, Surgery 2 (Subtotal or Near Total Thyroidectomy, or Removal of a Lobe and Partial Removal of the Contralateral Lobe) caused the highest cumulative incidence of DTC-related death.

## Data Availability

The raw data supporting the conclusions of this article will be made available by the authors, without undue reservation.
